# The Contribution of Spatially Tuned Brain Cells to Human Navigation Performance

**DOI:** 10.1007/s42489-026-00225-4

**Published:** 2026-08-17

**Authors:** Frank Dickmann, Annika Korte, Denise O´Meara, Cara Oster, Dennis Edler, Nikolai Axmacher, Julian Keil

**Affiliations:** 1 Geography Department, Cartography, Ruhr University Bochum, Bochum, Germany; 2 Department of Neuropsychology, Ruhr-University Bochum, Bochum, Germany

**Keywords:** Spatially tuned cells—grid cells—border cells, Head direction cells, Place cells, Navigation performance, Landmarks, Cardinal axes

## Abstract

Digital navigation systems facilitate goal-directed travel but reduce active engagement with the surrounding environment—a process critical for the formation of allocentric cognitive maps. Neuroscientific evidence reveals that spatial orientation relies on the coordinated activity of specialized spatially tuned neuronal populations, including place cells, head direction cells, grid cells, and border cells. This article introduces the specific properties of these spatially tuned cells, which encode central components of spatial information such as position, direction, distance, boundaries, and object relationships. Cell activities are continuously modulated by the perception of stable landmarks and environmental boundaries. This could form the basis for a “neurocartographic” approach that focuses on the systematic use of these cell activities to support spatial orientation. Enhancing the visual salience of landmarks and boundary structures, as well as integrating Virtual Reality (VR) and Augmented Reality(AR)-based components, may strengthen the metric representation of space—particularly grid cell–based coding. The overarching objective of this approach is to promote the development of allocentric and egocentric spatial representations during both real-world navigation and map-based wayfinding, thereby counteracting the decline in navigational abilities observed with the widespread use of digital turn-by-turn systems.

## Abstract (de)

Digitale Navigationssysteme erleichtern zwar das zielgerichtete Auffinden von Orten, reduzieren jedoch gleichzeitig die aktive Auseinandersetzung mit der räumlichen Umgebung – ein Prozess, der die Ausbildung allozentrischer kognitiver Karten wesentlich beeinträchtigt. Neurowissenschaftliche Befunde zeigen, dass räumliche Orientierung auf der koordinierten Aktivität spezialisierter Neuronenpopulationen im Gehirn beruht, darunter *Place Cells, Head Direction Cells, Grid Cells* und *Border Cells*. Der vorliegende Artikel führt in die spezifischen Eigenschaften dieser Zelltypen ein, die zentrale Komponenten räumlicher Information kodieren wie Position, Richtung, Distanz, Grenzen und Objektbeziehungen. Die Zell-Aktivitäten werden dabei kontinuierlich durch die Wahrnehmung stabiler Orientierungspunkte und Umgebungsgrenzen moduliert. Dies könnte die Grundlage für einen „neurokartographischen“ Ansatz bilden, der die systematische Nutzung dieser Zellaktivitäten zur Unterstützung räumlicher Orientierung in den Mittelpunkt stellt. Die gezielte visuelle Hervorhebung relevanter Orientierungspunkte und Grenzstrukturen sowie der Einsatz von Virtual Reality (VR) und Augmented Reality (AR)-basierten Elementen könnten die metrische Repräsentation der Umgebung – insbesondere die Gitterzellaktivität – stärken. Das übergeordnete Ziel dieses Ansatzes besteht darin, die Ausbildung allozentrischer und egozentrischer räumlicher Repräsentationen sowohl in realen Umgebungen als auch bei der kartengestützten Navigation zu fördern und so dem durch digitale Turn-by-Turn-Systeme beobachteten Rückgang der Navigationskompetenzen entgegenzuwirken.

## Introduction

**Fig. 1:**
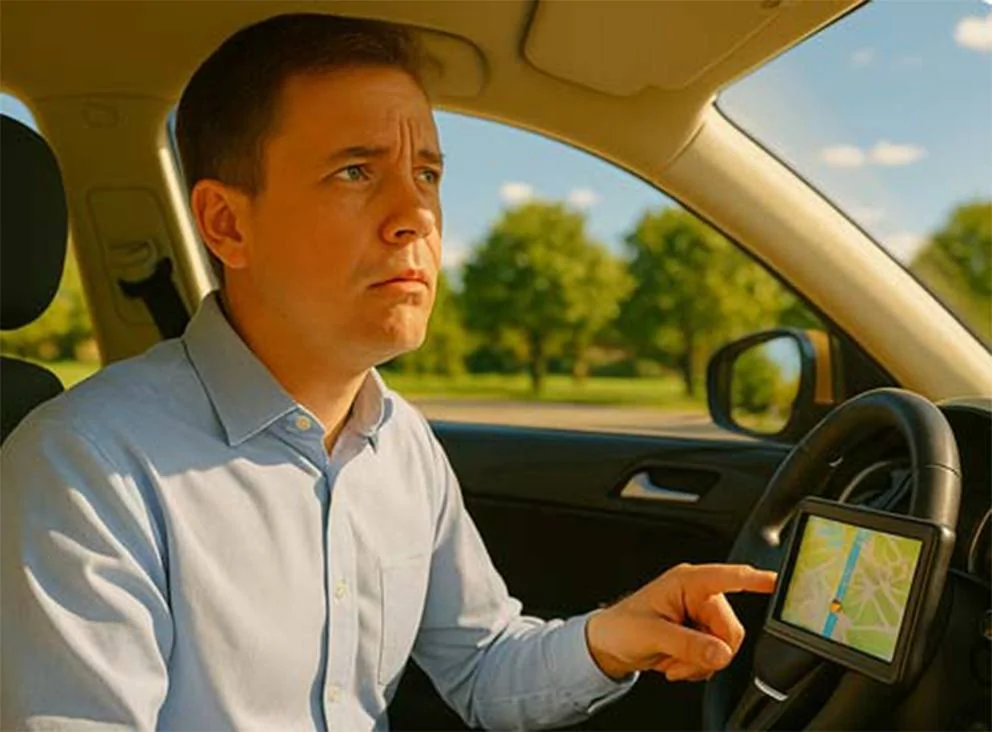
Relying on digital devices can impair orientation skills (AI generated picture, Dall-E)

Maps are used millions of times each day to help people orient themselves in space and navigate purposefully, whether by vehicle or on foot. Dynamic directional arrows on digital map displays, as well as augmented reality elements, now provide additional navigational support. In this article, the term “map” refers broadly to spatial information displays used in digital navigation systems, including mobile map interfaces, augmented reality guidance, and other visual spatial representations. With the ubiquity of mobile phones and digital navigation systems, printed maps have largely fallen out of everyday use. Today, the communication of spatial information required for navigation is often reduced to brief audio instructions (“Turn left in 200 m!”) or to visual arrows on a screen or head-up display. By simplifying navigation into arrows and short voice commands, these systems allow users to delegate most orientation tasks to the device. As a result, individuals are no longer required to actively orient themselves or to continuously verify their route against the surrounding environment (cf. Clemenson et al. 2021). Unlike traditional map reading, which required continuous cross-checking between map and environment, this active engagement largely disappears with navigation systems reducing the cognitive effort needed. Outsourcing these processes allows users to direct more cognitive resources to other tasks such as driving. However, it can reduce opportunities to train spatial memory and the interaction with the real environment. Instead, the short-term information provided by navigation systems through auditory turn-by-turn instructions or visual cues fails to contribute to higher-order spatial orientation (Ahmadpoor et al. 2020; Ben-Elia 2021; Ruginski et al. 2019). The “cognitive map” or “inner map” that emerges in the user’s mind while following digital navigation systems is therefore substantially reduced compared to the richer mental representation of space developed when using a printed map (Ying et al. 2024, Dickmann 2012). Currently there is no widely adopted solution that combines the practical advantages of digital navigation systems with measures to counteract the associated decline in spatial orientation skills. To mitigate such overreliance on technology or “technological infantilizing” (Montello 2009, p. 1835), the process of spatial information transfer through digital maps must be made significantly more effective (Fig. 1). A promising approach may lie in initiating an information transfer that does not rely on additional cognitive effort but instead builds on automatic neurocognitive processes in the user’s brain. Navigating through a (Virtual Reality (VR)) environment as well as reading a map activate neural processes in the brain that support spatial encoding.

Studies in cognitive psychology indicate that learning from maps leads to the acquisition of survey knowledge or spatial representations of an environment (Thorndyke and Hayes-Roth 1982). To gain a deeper understanding of the learning processes elicited by external (map-based) information that may be exploited for the design of navigation systems, the following section focuses on the role of neural structures in the brain. We assume that the neural systems involved in encoding the environment during spatial navigation (cf. Hafting et al. 2005) also contribute to the subsequent cognitive processing of spatial information (e.g., the acquisition of survey knowledge) and may therefore play an important role in map reading. This assumption is supported by findings from neuroscience demonstrating that imagining or remembering spatial scenes engages neural systems that substantially overlap with those recruited during direct experience of the environment through physical locomotion or eye movements (Bellmund et al. 2016; Horner et al. 2016).

The goal of the proposed “neurocartographic” approach (Dickmann et al. 2024) seeks to facilitate the neural processes involved in spatial representation by supporting the cell-based encoding of landmarks, environmental boundaries, and metric spatial relationships that are automatically engaged in the brain during spatial navigation. Advances in neuroscience in recent decades are providing increasing evidence of how spatial orientation processes are reflected in the firing patterns of spatially tuned cells in the human brain (Moser et al. 2015; Jacobs et al. 2013). Thus, navigation performance seems closely related to the activity of spatially coordinated cells (Epstein et al. 2017; Doeller et al. 2010). Since spatially oriented spatial cells can be addressed directly, it seems likely that improved navigational performance does not depend on any additional learning effort. This suggests that orientation based on spatial cells relies more on automatic processing than on deliberate learning. To improve navigation performance, research should be conducted to determine whether navigation systems could stimulate the neural networks in the human brain that are responsible for spatial orientation—whether visually or acoustically. Insights into the mechanism by which external stimuli can trigger the activity of spatially coordinated cells could therefore contribute to the development of innovative digital navigation systems.

The central argument of this paper is that recent findings from neuroscience can help to improve the design of digital cartographic interfaces. Rather than treating spatial cognition as a purely psychological process, we propose that map design can deliberately support the neural mechanisms underlying spatial orientation.

## External Activation of Neurons May Support the Formation of Spatial Representations

**Fig. 2:**
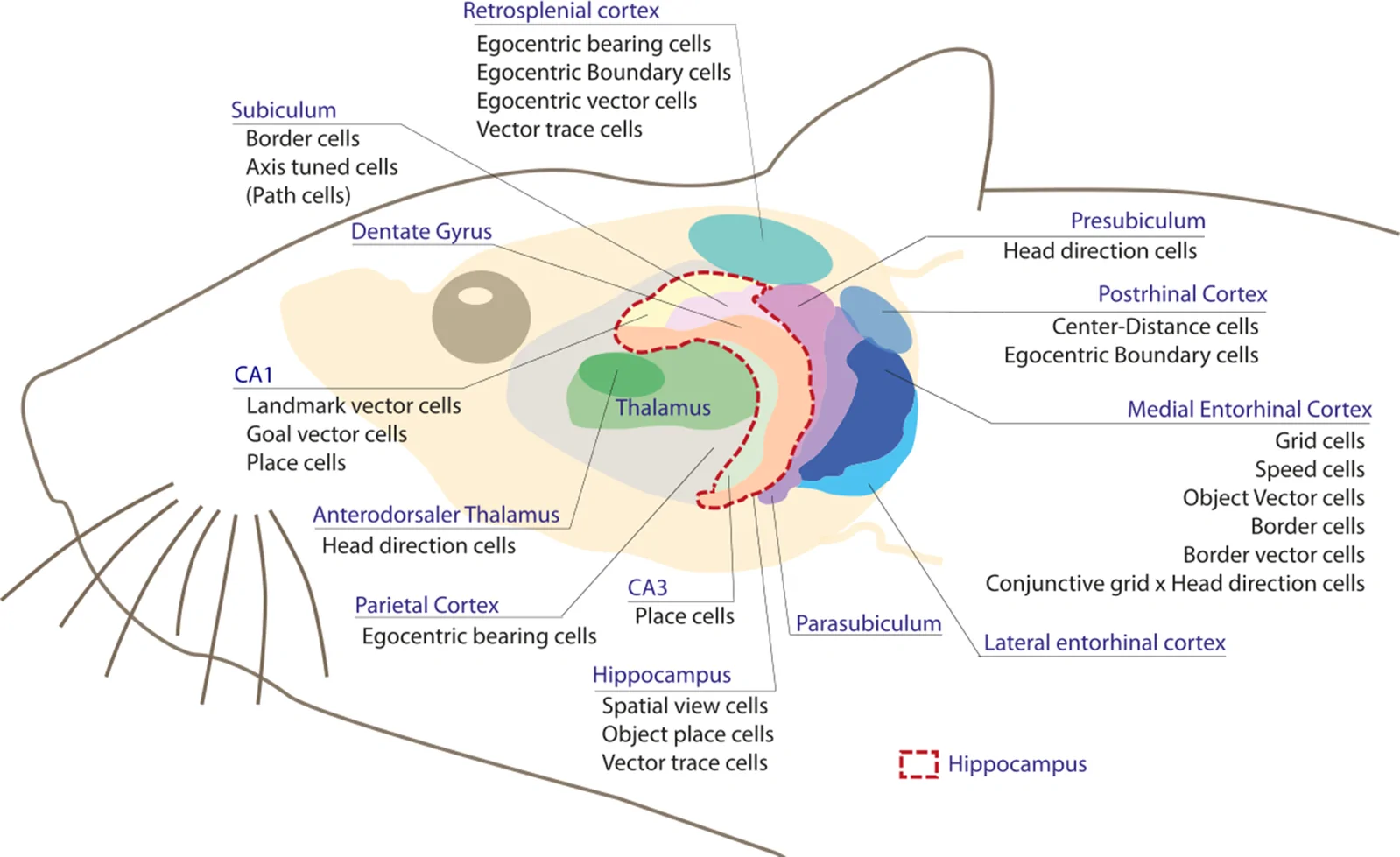
Spatially tuned cells in the brain. For illustrative purposes, the cells refer to the brain of a rat. Some cells have been found in humans or other mammals, e.g. bats, in comparable brain regions. The arrows do not indicate the exact position, but only the corresponding region of the brain (adapted and modified from Moser et al. (2014), with permission from Springer Nature, 6156680659679)

Although the neural mechanisms underlying spatial memory are still far from fully understood, research has demonstrated that groups of specialized cell types in the brain contribute to the internal representation of space (see Fig. 2; Hafting et al. 2005; Hartley et al. 2014; O’Keefe and Nadel 1978). The collective activity of these cells gives rise to a neural map of the environment (Moser et al. 2014). An individual’s location is thought to be encoded by the coordinated activity of multiple cell types, each characterized by distinct spatial firing properties, particularly within networks of spatially tuned cells in the hippocampus and adjacent entorhinal regions (Solstad et al. 2008; cf. Moser and Moser 2016). Over the past decades, animal studies have uncovered an increasing number of cells whose activity can be linked to navigational behaviour (Hafting et al. 2005; Kropff et al. 2015; Moser et al. 2014; O’Keefe and Nadel 1978). Sensory information about an environment is decomposed by the hippocampus into multiple context-specific representations (Hafting et al. 2005). This means, for example, that a spatial object such as a tree may be processed by one cell type, while a house wall behind it may be processed by another. The interplay of numerous active neural networks together generates an internal representation of space that serves as a “cognitive map” (Moser et al. 2015; O’Keefe and Nadel 1978) to guide movement, navigation and orientation. While most findings on cell activity come from rodent studies, there is growing evidence suggesting that the key spatial characteristics are also shared by other mammals, including humans (Hartley et al. 2014). Several Studies have identified neural correlates of higher-order navigation processes in humans (Doeller et al. 2010; Jacobs et al. 2013; Kunz et al. 2015), although the intensity of cell activity sometimes is less pronounced (Kunz et al. 2021).

Human spatial orientation relies on a complex system of interacting brain networks (Bicanski and Burgess 2018), which are the subject of ongoing neuroscientific research (Bierbrauer et al. 2020; Chen et al. 2018, 2024). External spatial stimuli modulate activity of neurons in the hippocampus and entorhinal cortex and establish a connection between neuropsychological processes and an individual’s movement through the environment (Buzsáki and Moser 2013). This suggests that external cues may be used to modulate spatial cell firing in a controlled manner via environmental cues. If we could address the activity of specific spatial cells with environmental stimuli, this might support the formation of representation of space (Dickmann et al. 2024). To achieve this, it is necessary to understand the firing characteristics of the relevant spatial cells and their interactions, which support spatial representation during navigation. A next step could be to trigger this cell activation through the perception of mediated spatial information, e.g., through maps or virtual/augmented reality techniques (Korte et al. 2024; Dickmann et al. 2024). The following section therefore introduces the properties of “space-responsive cells” (Derdikman and Moser 2014), “spatially modulated cells” (Moser et al. 2015) or “spatially tuned cells” (Gofman et al. 2019), a neuronal population crucial for the coding of self-localization and navigation, which has already been shown to respond to sensory (e.g., visual) environmental cues (Krupic et al. 2015; Navarro Schröder et al. 2020; Bellmund et al. 2016). These neurons are crucial for representing one’s position and guiding navigation (Solstad et al. 2008; Lever et al. 2009; O’Keefe and Nadel 1978). In addition, *speed cells* and *time cells* have been identified, responding to the temporal flow and velocity of self-motion through an environment (Eichenbaum 2014; Kropff et al. 2015; Moser and Moser 2016). Independent of movement direction, the firing rate of *speed cells* increases proportionally with the speed of locomotion (Kropff et al. 2015). *Time cells* exhibit sequential firing patterns that represent temporal intervals (Eichenbaum 2014). While *time cells* und *speed cells* do not directly encode spatial objects in the environment, the temporal information they convey is indispensable for representing movement through space and thus supporting navigation.

## The Role of Spatially Tuned Cells in Navigation

How different cell types interact to generate a cognitive map remains a key question in neuroscience (Long et al. 2025, cf. Moser and Moser 2008). Meanwhile, the catalog of neurons with distinct firing patterns directly implicated in spatial navigation continues to expand (Høydal et al. 2019) (see Fig. 2; Table 1).

**Table 1:** Neurons with spatial reference involved in the construction of the “inner map.” Each cell type encodes a specific aspect of the orientation process.

Cell	Brain Region(s)	Encoding of space	Firing Pattern	Study (year)
Place Cells	Hippocampus (CA1, CA3) (rats)	Body’s location in space	Fire when an individual is in a specific location	O’Keefe and Dostrovsky (1971)
Head Direction Cells	Postsubiculum, anterior thalamus, retrosplenial cortex (rats)	Gaze or body orientation in space	Fire only during movement in a specific direction	Taube et al. (1990)
Spatial View Cells	Hippocampus and parahippocampal gyrus (rhesus monkeys)	Viewed location in space, independent of position (alloc.)	Fire when looking at specific object of the environment	Rolls et al. (1997)
Grid Cells	Medial entorhinal cortex (MEC) (rats)	Covering surrounding surface	Fire regularly in a hexagonal pattern across space	Hafting et al. (2005)
Conjunctive Grid × Head-Direction Cells	Medial entorhinal cortex (deep layers)	Covering surrounding surface when looking in a specific direction	Fire at grid locations when moving in specific directions	Sargolini et al. (2006)
Border Cells	MEC, Subiculum (rats)	Proximity to walls or spatial boundaries	Fire close to an environmental border	Solstad et al. (2008)
Object-Place Cells	Hippocampus (rats)	Object (identity) + object location	Fire for specific object-location combinations	Komorowski et al. (2009)
Object cells (object-responsive neurons)	Lateral entorhinal cortex (LEC) (rats)	Object identity	Fire for the presence of a specific object (identity)	Deshmukh and Knierim (2011)
Landmark Vector Cells	CA1 (subgroup of place cells) (rats)	Individual landmark vector	Fire at specific direction and distance to a landmark	Deshmukh and Knierim (2013)
Speed Cells	Medial entorhinal cortex (MEC) (rats)	Movement speed	Fire proportionally to speed	Kropff et al. (2015)
Object-Vector Cells (OVCs)	Medial entorhinal cortex (MEC) (rats)	Single object vector	Fire at specific angle and distance to an object (without identity)	Høydal et al. (2019)
Vector Trace Cells	Hippocampus, retrosplenial cortex (rats)	Object location	Fire at previous object positions/own positions	Poulter et al. (2021)
Axis tuned cells (path cells)	Subiculum (rats)	Axes (paths)	Fire when at head orientations 180° apart	Olson et al.(2017)
Goal Vector cells (place cells subset)	CA1 (Hippocampus) (bats)	Vector	Fire when viewing to a goal	Sarel et al. (2017); Sarel et al. 2017
Border Vector Cells	Retrosplenial cortex (rodents)	Direction and distance to border	Fire at specific distance to a border	Bicanski & Burgess, (2020)
Egocentric Bearing Cells	Parietal cortex, retrosplenial cortex (humans)	Egocentric directions toward local reference points	Most cells fire at a specific angle to an object relative to the viewing position (some also fire at distances)	Kunz et al. (2021)
Egocentric Vector (Boundary) Cells	Postrhinal/retro-splenial cortex (rats)	Position of environ-mental boundaries relative to the animal (Vector: direction and distance)	Fire when there is a distant boundary/wall relative to an animal	Alexander and Nitz (2015), Park et al. (2024)
Center-Distance Cells	Postrhinal cortex (mice)	Center of an environment	Fire when approaching the center	LaChance and Taube (2023)

The activity of the various cell types can be differentiated according to distinct aspects of the orientation process. *Object cells* (or *object-responsive neurons*), for example, fire selectively in response to the immediate presence of a particular object (Deshmukh and Knierim 2011). In this case, the coding concerns the identity of a spatial object rather than its position within the environment. In contrast, other cell populations—such as the similarly named *object-vector cells*—represent the direction and distance to objects (i.e., spatial vectors), but not their identity or meaning (Høydal et al. 2019).

Neurons in the hippocampus respond complementarily to highly specific features of the environment (Gauthier and Tank 2018) and interact with other spatially tuned cells (Barry et al. 2006). The diversity of spatially tuned cells discovered so far—including highly specific cells such as *spatial view cells* (Rolls 2022, 1997), *vector trace cells* (Poulter et al. 2021), and *center-distance cells* (LaChance and Taube 2023)—thus reflects the many distinct components of the overall orientation process. Spatially tuned cells can be regarded as the building blocks of a network, which forms an “internal” representation of space. However, the classification of cells into clear categories is often difficult, as their firing can change under varying conditions (Deshmukh and Knierim 2013). Moreover, some spatially tuned cells encode multiple spatial properties simultaneously—so-called conjunctive cells (Sargolini et al. 2006; cf. Moser et al. 2014).

Despite this complexity, emerging evidence sheds light on the functional properties of spatially tuned neurons and their contributions to navigation and spatial orientation. To examine their contribution to allocentric, map-like representations of space, the following discussion centers on those neuronal populations whose characteristic firing properties are thought to play a pivotal role in the construction of allocentric spatial codes. These include place cells, head-direction cells, grid cells, and border cells (Hafting et al. 2005; Hartley et al. 2014; Lever et al. 2009; Solstad et al. 2008; Taube et al. 1990).

### Place Cells

**Fig. 3:**
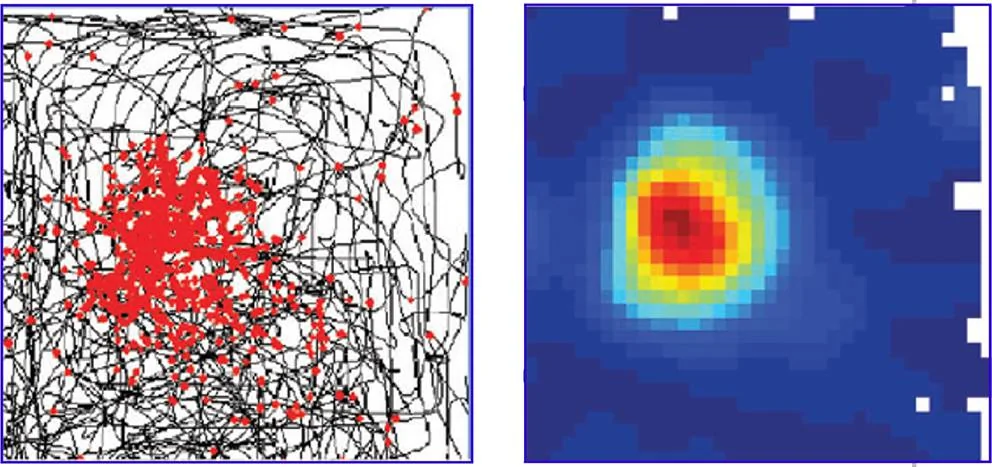
Left: The black lines show the trajectories of a foraging rat in a square enclosure. Spike locations of the place cell (place field) are overlaid in red on the trajectory. Each red dot represents a single spike of one single cell recording; right: Color-coded rate map (a two-dimensional autocorrelogram of the spatial rate distribution) with red indicating high activity and blue indicating low activity (Reproduced from Moser et al. (2015); with permission from Cold Spring Harbor Perspectives in Biology, Cold Spring Harbor Laboratory Press)

*Place cells*, first discovered in 1971 in rodents, are located in the hippocampus and fire selectively when an individual holds a specific location in the environment (O’Keefe and Dostrovsky 1971). Several decades later, they were also identified in humans (Ekstrom et al. 2003; Miller et al. 2013). The discovery of *place cells* (PCs) demonstrates how the brain constructs an abstract, cognitive representation of space (Grieves and Jeffery 2017). Recordings from *place cells* in foraging rats showed peak activity when the animal’s head was within a particular area of the environment—regardless of the direction it was facing. This area of increased activity is referred to as the cell’s place field (O’Keefe 1976; O’Keefe and Nadel 1978). Outside this specific place field, little to no activity occurs in this specific place cell (see Fig. 3). Each *place cell* fires at a distinct location. When the animal moves to a new position within the environment, a different place cell becomes active. It is generally assumed that the entire surface of an environment is represented by the combined activity of the population of *place cells* (Grieves and Jeffery 2017). However, locations are not represented in the brain in the same way as on a topographical map: two *place cells* associated with two neighboring positions in real space are not necessarily next to each other in the hippocampus.

*Place cell* activity is experience-dependent and emerges through learning processes during the exploration of an environment (Cohen et al. 2017; Wilson and McNaughton 1993). However, these cells also fire in the absence of visual perception. Firing can be based solely on internal signals from the vestibular system, self-motion, and running speed (Sharp et al. 1995). They remain active even when an individual moves in complete darkness. The firing patterns of *place cells* in the hippocampus are therefore closely tied to the spatial representation of an environment, but they are modulated by diverse sensory inputs, such as visual, auditory, or tactile (Knierim et al. 1995). *Place cells* in the hippocampus fire when an individual is at a particular location, but they can also reactivate at positions that the individual has visited in the past (Hartley et al. 2014; Moser et al. 2015; O’Keefe and Dostrovsky 1971). When entering an environment—even a familiar one—*place cells* are activated or reactivated based on the arrangement, combination, and relative distances of objects and, above all, landmarks (Knierim et al. 1995). Hence, multiple cues must converge for *place cells* to fire (Deshmukh and Knierim 2013; O’Keefe and Conway 1978; O’Keefe and Speakman 1987). Visually perceived objects or landmarks act as external reference points that reactivate and stabilize the firing pattern (place fields) of *place cells* (Knierim et al. 1995). Subsets of them were also observed to respond specifically to nearby objects. These so-called *landmark vector cells* (SVC) fire in relation to a specific distance and direction from a particular object identity. In this case, the individual must be located at a defined position relative to the object (Deshmukh and Knierim 2013).

A more allocentric character is exhibited by the recently discovered subgroup of *place cells* known as *object vector cells* (OVCs). OVCs fire when an (unspecified) object is located at a particular distance and direction relative to the individual’s position (Høydal et al. 2019). In contrast to *place cells* or *landmark vector cells,* which respond to the identity of objects, OVCs encode distance and direction (i.e., vectors) to objects independent of identity. While they do not directly represent an absolute location, OVCs provide essential vector information that allow for the positional determination of objects relative to the individual’s own position within an environment.

Because the information represented by *place cells* refers to specific contexts of a single spatial scene, they cannot provide overarching spatial relations to objects in previously or subsequently visited different environments (Julian and Doeller 2021). For example, distances between locations belonging to different environments cannot be inferred. When entering a new environment, a *place cell* alters its firing behavior completely, a phenomenon known as “remapping” takes place (Grieves and Jeffery 2017). The same *place cell* that fires at one location in one environment will fire at a different location in another. *Place cells* encode locations in relation to the relative position of objects, but not with respect to an absolute coordinate system. Thus, while a *place cell* points to a single location within a spatial scene, it does not generate an overview of the broader spatial situation (Eichenbaum et al. 1999). The firing of a *place cell* does not reveal where the represented location lies in relation to a neighboring or higher-level spatial unit—for instance, within a region or a country. Although the activity of spatially tuned cells is sometimes described as forming “maps” (Moser and Moser 2016), *place cells* in fact produce only multiple independent spatial representations (“fragmented sub maps”) (Derdikman and Moser 2010). While *place cells* encode allocentric information within a given scene (such as the configuration of nearby objects), their activity alone is insufficient to generate a higher-order representation of space in the sense of a unified “master map” that extends beyond a single scene. Integrating codes are necessary to link the separate maps with each other (Morton et al. 2017, cf. Ottink et al. 2025 for hippocampal integration of distances).

Further studies have revealed that *place cells* respond not only to the presence of spatial objects (distal landmarks) and to changes in environmental geometry (Leutgeb et al. 2005; Park and Lee 2016), but also to non-spatial inputs. Studies with rats by Save et al. (2000) have demonstrated that the stability of *place cell* activity can be modulated by sensory influences such as odors. This suggests that the activation of *place cells* can also be supported by object properties (e.g., size, salience) and their context (e.g., environmental smells, sounds, or task relevance) (cf. Komorowski et al. 2009). Omer et al. (2018) have shown that populations of *place cells* can also be activated when the position of a conspecific is observed (“*social place-cells*”). *Place cells*, therefore, appear to represent both spatial and contextual (semantic) aspects of an environment (Eichenbaum et al. 1999; Miller et al. 2013). This dual coding underlines their role in navigation, since successful self-localization depends not only on the relative position of objects but also on their meaning or identity (Eichenbaum et al. 1999). Linking object position with object identity is particularly crucial for the map-based communication of spatial information (Dickmann 2018, p. 126). Consequently, *place cells* may serve an important function in externally guided interventions aimed at influencing spatial orientation.

In sum, *place cells* are not merely the end point of upstream neural processing; they form part of a broader network that supports spatial orientation. Effective orientation relies on the combined input of multiple spatially tuned cell types (Deshmukh and Knierim 2013). Research has shown that *place cell* firing is shaped by the concurrent activity of other spatially responsive neurons, including head-direction cells (Taube 2007) and grid cells (Moser et al. 2008; 2015). Place fields therefore reflect higher-level spatial constructs that arise from the integration of more fundamental components, such as directional cues, environmental boundaries, and self-motion information (Grieves and Jeffery 2017).

### Head Direction Cells

*Head direction cells* (HDCs) were first discovered in the mid-1980 s in rodent experiments within the postsubiculum, a region located between hippocampus and entorhinal cortex (Boccara et al. 2010; Taube et al. 1990; Taube 1998). Head-direction-like neural activity has also been observed in humans (Baumann and Mattingley 2010; Jacobs et al. 2010). Similar to *place cells*, the stability of HDC firing depends on the perceived stability of environmental cues (landmarks) (Knierim et al. 1995). Unlike *place cells, head direction cells* do not encode spatial positions but directional information, or azimuth (Whitlock and Derdikman 2012). These directional signals are regarded as a “key aspect of navigation for any organism” (Page and Jeffery 2018).

**Fig. 4:**
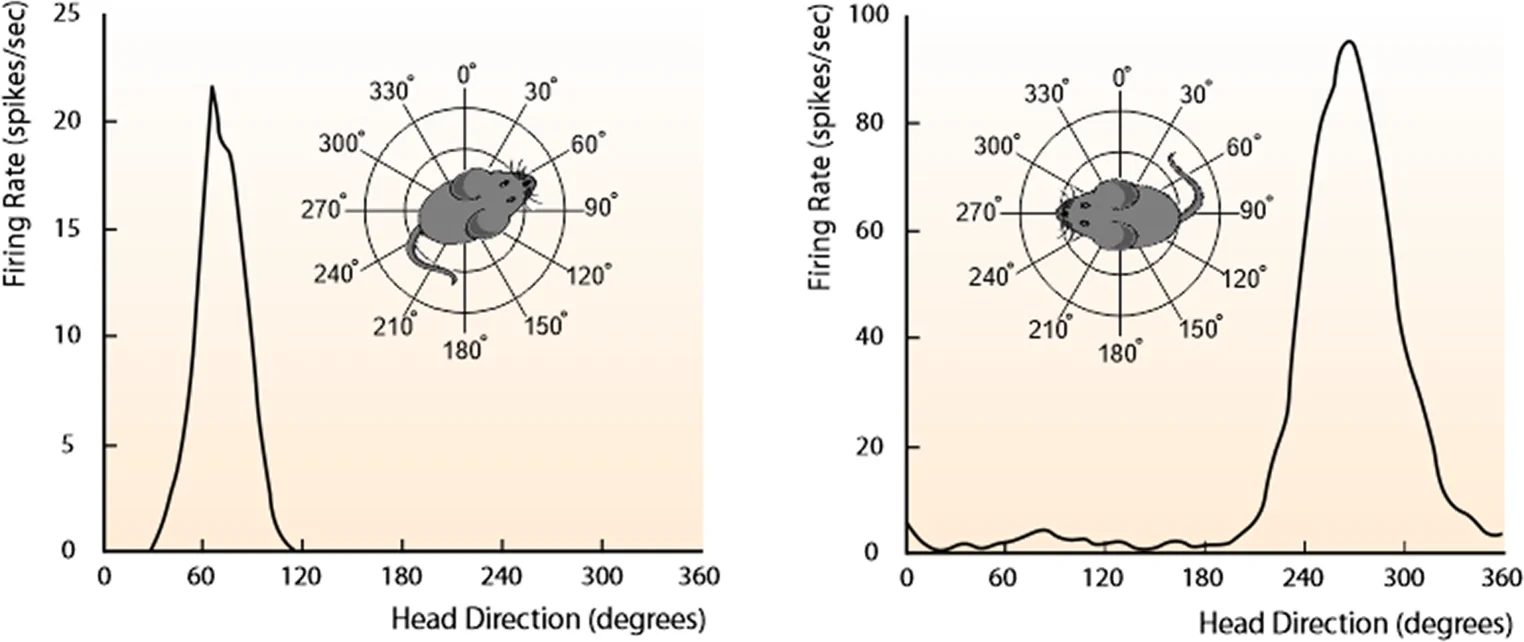
Activation of head direction cells in rodents; depending on the direction of the head, strong activation occurs (here using the example of two head direction cells) (Reproduced and modified from Taube et al. (1990), with permission from JNeurosci/Society for Neuroscience)

HDCs fire when an individual’s head turns toward a specific direction, regardless of the current location. Within an environment each HDC exhibits a preferred firing direction (Lozano et al. 2017) (Fig. 4). This cell type is essential for continuous orientation, as it encodes the head position of the individual relative to the surrounding environment (Taube et al. 1990). When the individual turns to face another direction, a different group of HDCs becomes active. Correct registration of head rotations relative to the initial orientation is supported by vestibular self-motion signals from the inner ear (Blair and Sharp 1996, Yoder and Taube 2014). Across the population of HDCs, the entire 360° range is represented (Dudchenko et al. 2019). Importantly, their coding is not relative to the body’s orientation but to the perceived environment. HDCs thus operate like a “compass” that aligns with the external world (Knierim 2005). This property makes them particularly important for navigation, as they provide a stable reference to the environment. This distinguishes them from the more recently identified *egocentric bearing cells* (EBeCs) in humans (Kunz et al. 2021), which encode direction relative to one’s own position and may thus bridge egocentric and allocentric representations.

Although robust even in the absence of visual input, HDCs rely on stable environmental references to maintain long-term accuracy (Knierim et al. 1995). HDCs remain active in darkness or when landmarks are removed, though their firing becomes increasingly error-prone without external correction (Knierim et al. 1995; Muller et al. 1996). Upon entering a novel environment with new landmarks, HDCs rapidly realign to the updated reference frame (cf. Dudchenko et al. 1997). While the HDC network can be maintained through self-motion cues alone (Tan et al. 2015; Yoder and Taube 2014), its sensitivity to external anchoring enables the formation of a stable internally maintained directional system. This means that prominent landmarks—such as tall buildings or mountain peaks—could provide strong anchoring cues for navigation. The population of HDCs contributes directional information to a coherent spatial representation and is closely interconnected with *place cells *and *grid cells* (Knierim et al. 1995; Taube 2007; Whitlock and Derdikman 2012).

### Grid Cells

**Fig. 5:**
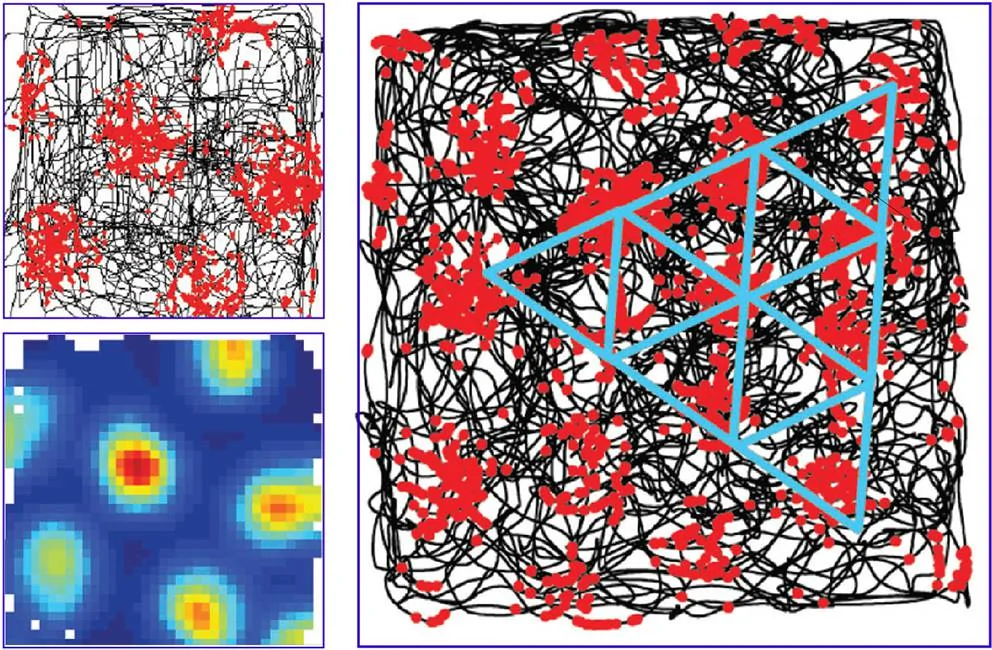
Regular hexagonal firing pattern of grid cells (entorhinal cortex); left: the black lines show the trajectories of a foraging rat in a square enclosure with a diameter of 1.5 m. Spike locations of the grid cell are overlaid in red on the trajectory. Each red dot represents a single spike of a grid cell; color-coded rate map (a two-dimensional autocorrelogram of the spatial rate distribution) with red indicating high activity and blue indicating low activity. The firing fields of grid cells exhibit a repeating pattern of equilateral triangles that together form a consistent hexagonal grid regularly tiling the entire environment (Reproduced from Moser et al. (2015); with permission from Cold Spring Harbor Perspectives in Biology, Cold Spring Harbor Laboratory Press)

*Grid cells* were first identified in the medial entorhinal cortex of rats (Hafting et al. 2005; Fyhn et al. 2004). Using magnetic resonance imaging (fMRI), evidence for grid cells—often referred to as “grid cell-like representations”—has also been found in humans (Doeller et al. 2010; Kunz et al. 2015; Jacobs et al. 2013; Nadasdy et al. 2017). *Grid cells* (GCs) exhibit a distinctive firing pattern: their activity fields form a repeating lattice of equilateral triangles (Derdikman and Moser 2014; Hafting et al. 2005) (Fig. 5). The activation of *grid cells* and the emergence of their firing fields are driven by an individual’s movement through space (Doeller et al. 2010), supported by vestibular signals and proprioceptive feedback from self-motion (RG et al. 2025). As a result, *grid cells* can continuously update positional information even in the absence of external sensory cues, such as in complete darkness (Hafting et al. 2005; Moser and Moser 2008). They are therefore thought to form the core of an intrinsic positioning system in mammals, providing a foundational mechanism for spatial navigation (Buzsáki and Moser 2013; Fiete et al. 2008; McNaughton et al. 2006).

In a novel environment, *grid cell* firing is initially irregular (Hafting et al. 2005). However, when salient boundaries are present, the hexagonal pattern rapidly aligns with these environmental features—supported in part by head-direction signals (McNaughton et al. 2006)—and subsequently stabilizes (Derdikman et al. 2009; Krupic et al. 2015; Stensola et al. 2015). Animal studies (in rats) demonstrate that this alignment persists when boundaries are shifted or rotated (Hafting et al. 2005). Like *head-direction cells, grid cells* provide a stable internal framework for the accurate representation of space. The consistent spatial alignment of the grid allows an organism to determine its position—and the distances between positions—relative to external reference points such as landmarks or boundaries, a prerequisite for navigation and path integration (Rowland et al. 2016). The regularity of the hexagonal pattern likely supplies the essential information required to estimate distances between one’s own location and an object, as well as between objects within the environment (Schøyen et al. 2025).

#### *Grid Cell* Geometry

**Fig. 6:**
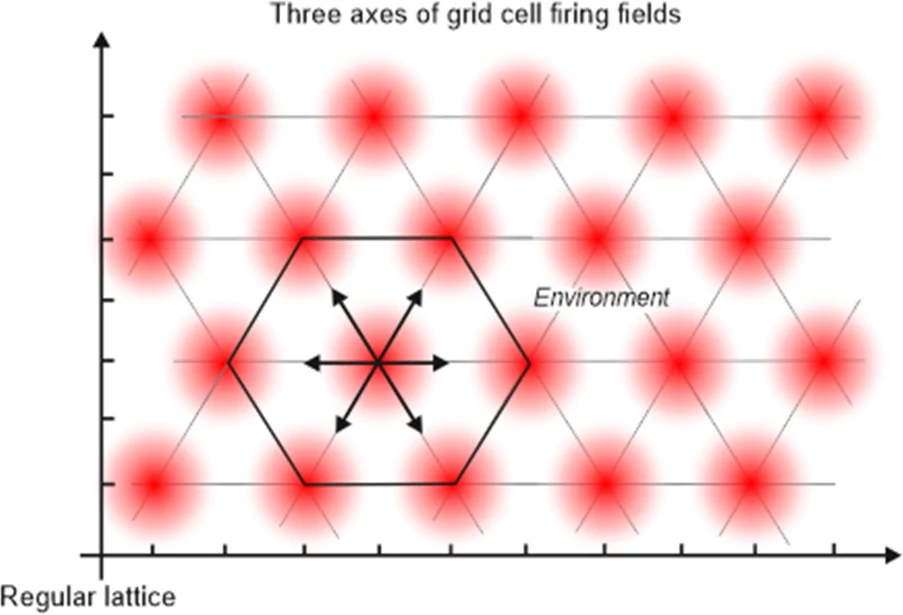
Grid cell activity mirrors a stable internal metric (map) of the environment

The discovery of *grid cells* spanning space as the “microstructure of a spatial map” in the brain (Hafting et al. 2005) marked a decisive step toward understanding human navigation. Unlike *place cells*, which have a single (or few) firing field(s), each *grid cell* has multiple firing fields (Moser et al. 2015, Hafting et al. 2005). A single *grid cell* is activated at several evenly spaced locations within the environment. Each grid cell becomes active as soon as the individual moves into those locations. Collectively, these firing fields form a repeating array of equilateral triangles that tessellate the environment into a continuous hexagonal grid (Derdikman and Moser 2014; Hafting et al. 2005; Moser et al. 2008). In contrast to place cells, which encode individual locations within specific environments, the *grid cell* pattern provides a universal lattice. It functions as a kind of internal longitude-latitude system, covering the entire environment in which an animal or human is situated with receptive fields (Moser and Moser 2016). The structured arrangement of grid fields—with their axes and characteristic regular spacing—may provide a neural basis for estimating direction and distance, enabling the brain to compute both the extent and the direction of movement through space (Welinder et al. 2008). The striking regularity of the hexagonal lattice can thus be interpreted as the geometric scaffold of an internal spatial map (Fig. 6).

Ensembles of *grid cells* maintain fixed spatial relationships. Within the same region of the entorhinal cortex, the offset between the grid patterns of two cells remains constant across different environments. This rigidity indicates that the grid cell network does not encode environmental context but instead provides a basis for precise positional information relative to external reference points (Moser and Moser 2008). Although the emergence of the grid cell firing pattern is not caused by external surroundings, geometric boundaries and environmental axes play an important role, as they influence the orientation of the grid’s hexadirectional axes and function as an anchor to stabilize grid cell firing (Keinath et al. 2018, Krupic et al. 2015). Thus, the grid structure is generated internally but calibrated externally. This external calibration opens a window for the controlled modulation of grid structure from outside.

#### Remapping

The stable alignment of *grid cell* patterns to environmental boundaries (Krupic et al. 2015) and landmarks (Savelli et al. 2017) supports the construction of an allocentric representation of space. Through the stable alignment of the grid, the brain has access to a two-dimensional metric reference system (cf. Moser and Moser 2008, Hafting et al. 2005).

**Fig. 7:**
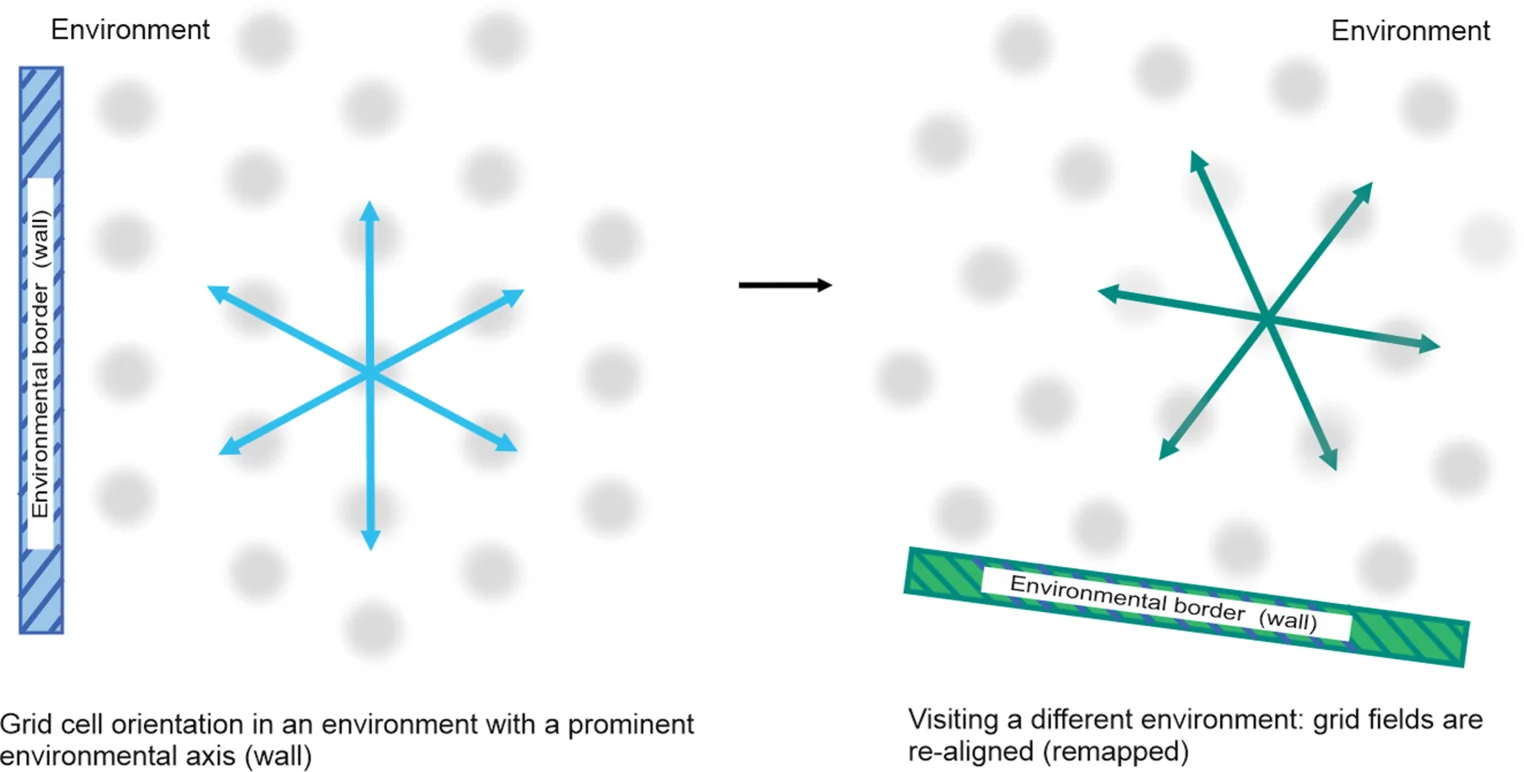
Alignment of grid firing fields (grey) within an environment according to a dominant environmental axis (blue wall/boundary). When entering (black arrow) new environments, the grid fields rotate to align with new, distinctive environmental boundaries (green wall)

When entering a different environment, changes in environmental geometry and the configuration of salient boundaries can lead to a reorganization of the grid-cell representation (Fig. 7). In such cases, the orientation and phase of the grid may realign to the spatial reference frame of the new environment, which is defined by its geometry, boundaries, and stable landmarks, before stabilizing again (Derdikman et al. 2009; Julian and Doeller 2021).

This provides a renewed geometric framework that can be used to reference the spatial content of a new environment. In this way, the characteristics of different spaces are encoded separately, reducing potential memory interference (cf. Colgin et al. 2008; Hafting et al. 2005; Fyhn et al. 2007). Depending on the shape of the environment (e.g., in narrow spaces), however, distortions of the geometric grid pattern may occur (Chen et al. 2024; Ginosar et al. 2023; Krupic et al. 2015; Stensola et al. 2012). Such destabilizing processes are likely to impair orientation (cf. Bellmund et al. 2020).

**Fig. 8:**
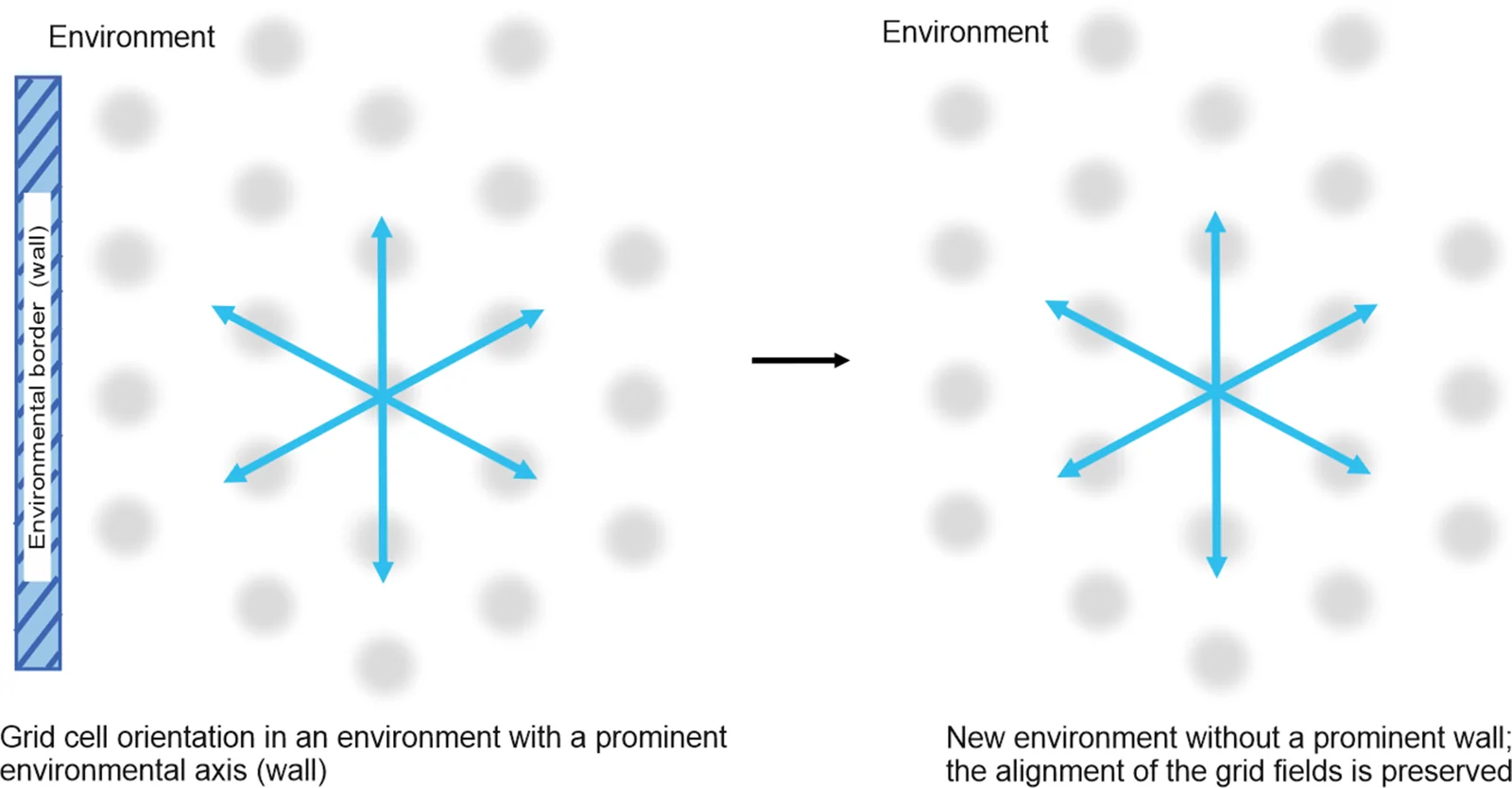
Grid firing fields (grey areas) aligned and stabilized along the boundaries (walls) in an environment; when entering a new environment without a prominent environmental axis, the original alignment is transferred (cf. Carpenter et al. 2015)

Another defining feature of *grid cells* is their ability to maintain firing even when reference points are no longer present (Hafting et al. 2005; Moser and Moser 2008). In other words, not every change of perspective triggers a dissolution and remapping of the grid cell pattern. This has been shown in animal experiments in which boundaries of the experimental environment were altered or rotated (Hafting et al. 2005). Accordingly, the positional relationships among *grid cells* can persist even in darkness or after an environmental change (Fyhn et al. 2007, Moser et al. 2014). Experiments with rats further demonstrated that the sequence of cells firing is repeated when the animal moves in the same direction within an adjacent space (Moser and Moser 2016). Carpenter et al. (2015) observed that when animals entered a new but connected environment, the firing of *grid cells* aligned with the orientation established in the previous compartment. This adjustment resulted in a single, coherent grid representation spanning both environments, enabling locations in the different compartments to be placed into a common metric framework (Fig. 8).

**Fig. 9:**
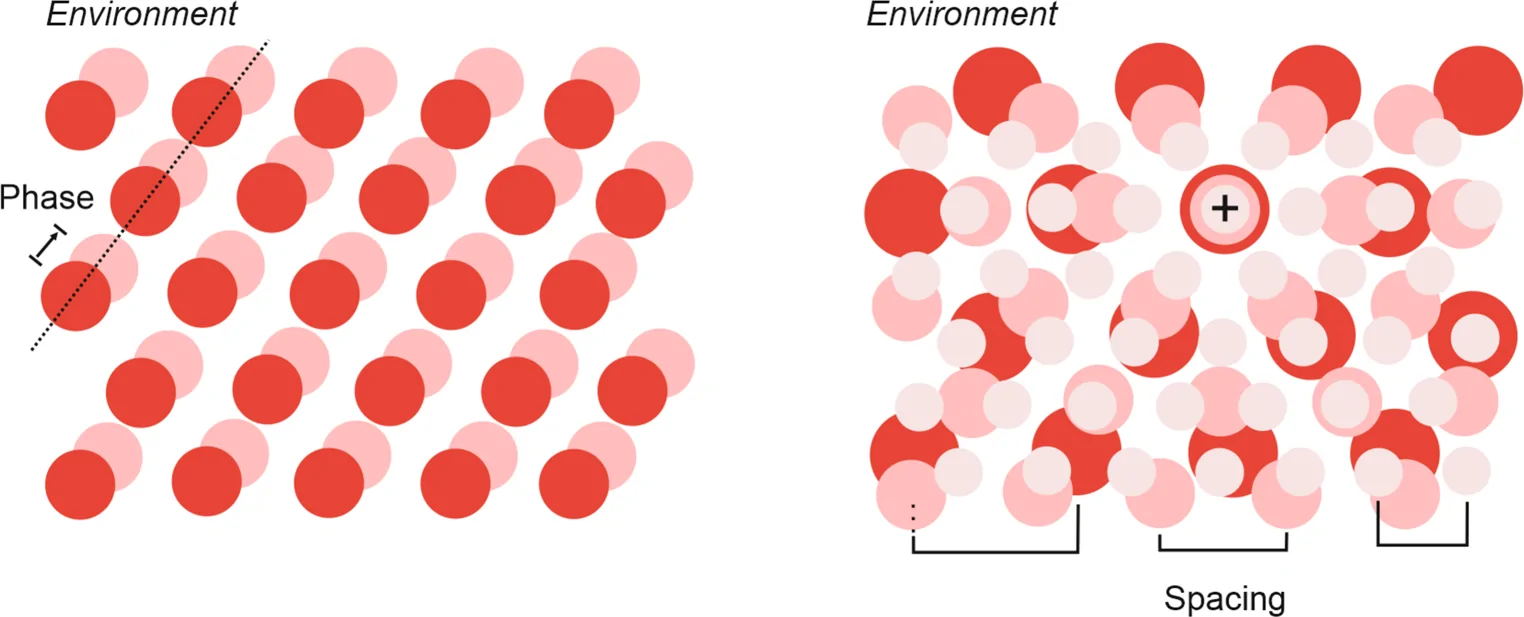
Left: Two adjacent grid cell firing fields (of one module) covering the environment—slightly shifted along one of the hexadirectional axes (phase); right: multiscale coding by three different modules of grid cells (with different spacings and sizes of firing fields); the maximum interference pattern probably leads to position coding. Within this environment a completely overlapping, i.e. combined activity, only occurs at a very specific place (black cross) (cf. Stemmler et al. 2015)

Translated to human navigation, this likely means that grid alignment is preserved (i.e., no remapping occurs) when consecutively visited environments share a highly uniform character, such as homogeneous steppe landscapes or forest areas without salient reference points or boundaries. In such cases, multiple environments are integrated into a common representation (Carpenter et al. 2015, cf. Savelli et al. 2017). The existing grid orientation is carried over into adjacent environments, thereby providing a foundation for overarching estimates of direction and distance (see Fig. 9). For navigation over long distances, where the aim is not to represent every subspace in detail but rather to maintain continuous alignment with a distant target, stable alignment of the *grid cells* appears to be more suitable. This is particularly relevant for constructing coherent spatial representations of large-scale environments. It can be assumed that frequent remapping in such cases will tend to be disadvantageous.

#### Positions and Distances

Another characteristic of *grid cells* is that neighbouring cells often share identical grid orientations and scales. They differ in their precise location of the grid nodes, which results in a slight offset of the grid fields in phase along the hexadirectional axes of the geometric lattice (Hafting et al. 2005). As a result, the intermediate spaces-those not covered by the receptive fields of a single cell—are also encoded (see Fig. 9, left). With the grid pattern of a single grid cell, a specific position (e.g., one’s own location) cannot be directly determined, since a single cell exhibits activity at multiple, periodically repeating locations (Hafting et al. 2005). This precludes unambiguous localization of a place—or of one’s own position—in space. Nevertheless, the regularity of grid cell firing fields can be harnessed for precise position coding. Modelling studies have shown that, in combination with the activity of additional grid cells, unique localization becomes possible (Solstad et al. 2006; Stemmler et al. 2015, cf. Schøyen et al. 2025).

The key lies in distinct populations of grid cells (modules) arranged along the dorsoventral axis of the medial entorhinal cortex (Hafting et al. 2005). These modules contain *grid cells* with different grid spacings and field diameters (sizes), whose activity patterns also overlap (Brun et al. 2008). This modular organization enables a multiscale coding of space. It is assumed that spatial environments are represented at different resolutions: fine-grained arrangements (with smaller grid-node spacing) encode local areas, while larger spacings are used for extended environments (Brun et al. 2008; Derdikman and Moser 2014; Hafting et al. 2005). The integration of signals from multiple *grid cells* with different phases and grid scales generates interference patterns, whose combined activity reaches a maximum at only one specific location in the environment (see Fig. 9, right). At this point of strongest overlap, many cells fire synchronously at a high rate, producing a particularly salient neural signal (Fyhn et al. 2007, Hafting et al. 2005). This convergence appears to enable unambiguous, high-resolution positional coding. Since *grid cells* differ in their spacing, each location within the local environment is associated with a unique combination of active cells. Neurons with access to this combined activity can therefore reliably determine one’s position (Moser et al. 2014). Mathematical models further show that the overlap of grid fields with different spacings arranged in a geometric progression with a 3:2 ratio can yield precise estimates of both position and distance (Stemmler et al. 2015). These predictions are largely consistent with empirical findings on the variability of grid spacings in rodents (Hafting et al. 2005). It is assumed that the activity generated by *grid cells* induces strong synaptic excitation in downstream neurons, particularly hippocampal *place cells* (Moser et al. 2008; Solstad et al. 2006). Other reseachers argue against such a strictly hierarchical view, instead emphasizing the role of sensory environmental inputs in driving *place cell* activity (Morris and Derdikman 2023) or assigning greater importance to *place cells* themselves in storing positional information (Rennó-Costa and Tort 2017). Nevertheless, even within these perspectives, *grid cells* are attributed a central role in stabilizing and maintaining place cell activity. The potential interactions between *place cells* and *grid cells* therefore support the idea that external references can exert a significant influence on navigational ability (Dickmann et al. 2024). The responsiveness of *grid cells* to sensory environmental inputs highlights the possibility of externally modulating their firing behavior. Although the geometric regularity of the hexagonal *grid cell* firing pattern is primarily the result of preprocessed neural signals from intrinsic brain circuits (RG et al. 2025; Hasselmo 2008); their firing patterns clearly reveal the influence of sensory (especially visual) signals from the environment (Bellmund et al. 2016; Krupic et al. 2015; Solstad et al. 2008).

### Border Cells

**Fig. 10:**
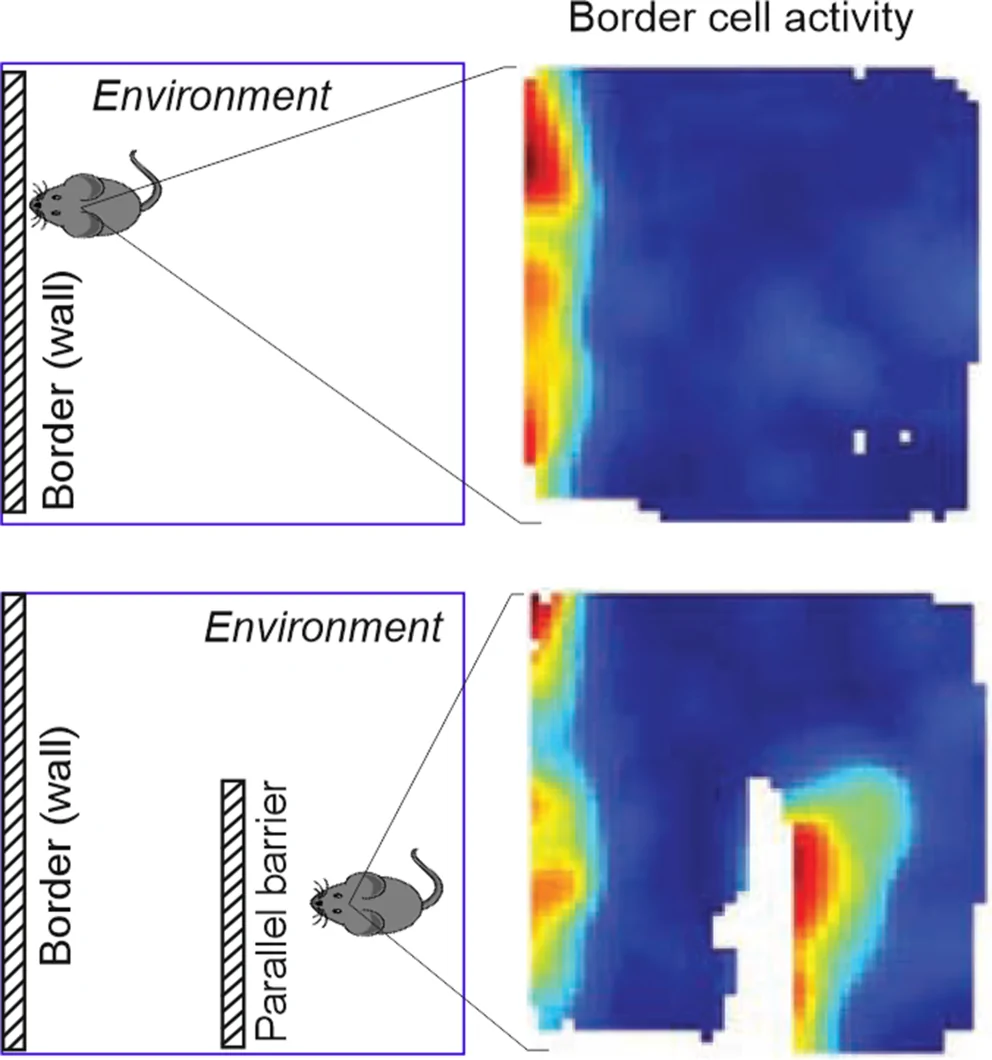
Activation of a single border cells (of a rat) through the experience of walls (borders/boundaries) in a square environment (below: spatially tuned cell with two active fields; (adopted from Solstad et al. 2008, rate map; with permission, Order Nr 5996530190102))

Another prominent cell type that responds directly to environmental stimuli are *border cells*. First identified in the entorhinal cortex of rats in 2008, these specialized neurons fire when an individual is positioned near a boundary of the environment (Solstad et al. 2008; see also Lever et al. 2009; Savelli et al. 2008) (Fig. 10). Within a given environment, different *border cells* are activated at specific borders (boundaries). Evidence for *border cell*-like activity has also been reported in humans (Stangl et al. 2021; Lee et al. 2018; cf. Yoo et al. 2022, on *episodic boundary cells*).

*Border cells* exhibit firing fields that align with specific geometric boundaries of the proximal environment, independent of the boundary’s length (Solstad et al. 2008). The cell populations that respond to borders/boundaries are not homogeneous: “classical” *border cells* are active when an individual is located in close proximity to a boundary (Solstad et al. 2008), whereas *“boundary” *(or *“border”) vector cells* (BVCs) have receptive fields and fire at specific distances and directions to a boundary (Bicanski and Burgess 2020). BVCs thus encode vectors, representing both direction and distance to a boundary (Lever et al. 2009). When direction or distance exceeds the tolerance range of a given BVC’s receptive field, a different BVC becomes active (Bicanski and Burgess 2020). Similar to place fields, the receptive fields of these vector-coding cells are independent of the animal’s orientation, but they shift in alignment with dominant environmental cues that also influence head direction cell activity (Bicanski and Burgess 2020). The combined activity of numerous cells, each responding to different distances and directions, produces a coherent spatial representation.

**Fig. 11:**
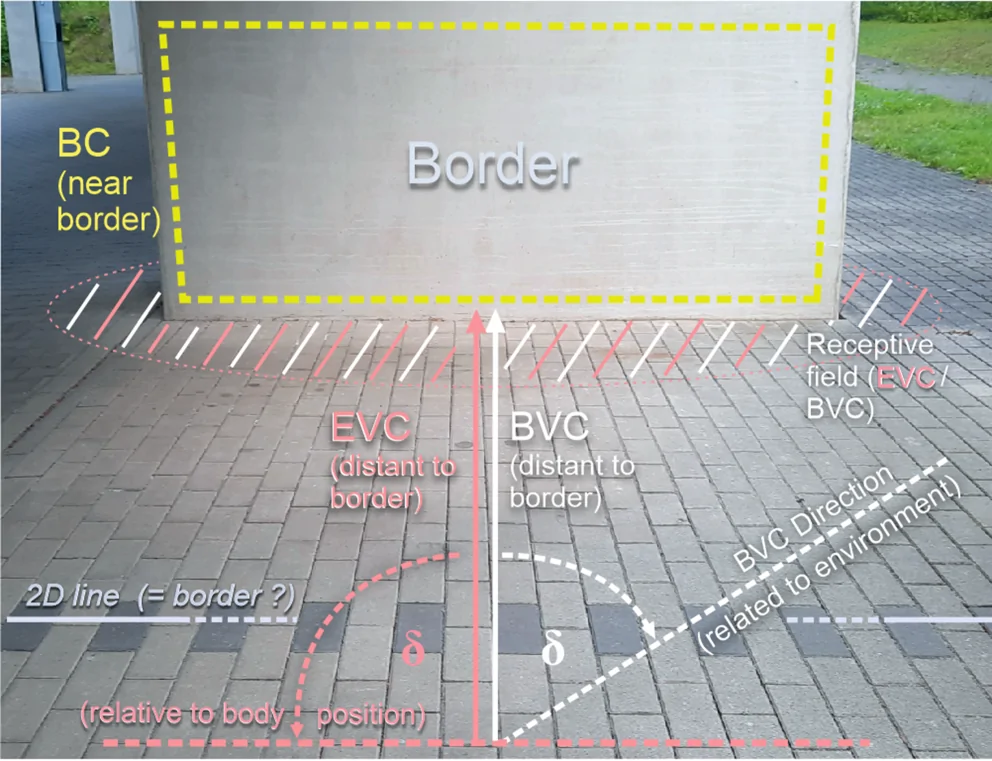
*Border cells* (yellow) become active in close proximity to a border/boundary (wall); *boundary vector cells* (BVC, white) represent a vector (distance and direction) to an environmental boundary (cf. Bicanski and Burgess 2020), *egocentric vector cells* (red) encode the direction and distance of a boundary relative to the body position (Alexander et al. 2020)

Since boundaries act as fixed positions in the environment, *border cells* play a central role for the construction of a mental representation of space. They can define the extent of an environment and serve as a reference frame for place representations (Alexander et al. 2020; Solstad et al. 2008) (Fig. 11). Likely in interaction with grid cells, distances and positions relative to boundaries can be determined (Moser and Moser 2016). *Border cell* types may therefore be crucial for anchoring the grid pattern to the geometric limits of the environment (Moser and Moser 2008; cf. Pollock et al. 2018).

*Egocentric vector (boundary) cells* (EBCs) play a key role in converting egocentrically perceived information into allocentric representations (Alexander et al. 2020; cf. Wang et al. 2018; see also *egocentric bearing cells* (EBeCs) found by Kunz et al. (2021) encoding only direction). Similar to allocentric *border cells* (BCs) and *boundary vector cells *(BVCs), the activity of *egocentric boundary cells *(EBCs) is linked to environmental boundaries. However, activation here occurs relative to body orientation rather than to the external environment. EBCs do not encode the absolute position of a boundary in space (e.g., a wall to the north), but rather its relative position with respect to the body (e.g., a wall in front of the individual) (Alexander et al. 2020; Gofman et al. 2019; Hinman et al. 2019).

Animal experiments show that the activity of the different *border cells types* is not limited to surrounding walls, but can also be triggered by smaller barriers (Lever et al. 2009; Solstad et al. 2008). *Border cells* even respond to minimal changes in terrain and, importantly, regardless of the color, material, or shape of the boundary (Bicanski and Burgess 2020; Lever et al. 2009). Thus, environmental boundaries that trigger the activation of boundary cells can take many forms. Since even small, passable gaps in the ground can trigger cell activity (Lever et al. 2009), it is conceivable that even simple visual boundaries, such as lines on the ground, could act as potential boundary signals for border cells (see Fig. 11 below). Some researchers understand a border as a trigger for border cell activity not only as a physical structure, but increasingly as an abstract construct that is already determined by the visual perception of a border (presence) (Lever et al. 2009; Moser and Moser 2008; cf. Yoo et al. 2022). The versatility of boundary-related stimuli and the persistence of border activity (Bicanski and Burgess 2020) suggest that boundary vector cells play a key role in linking external cues with neural processes of spatial orientation.

## The Construction of a Cognitive Map During Navigation

**Fig. 12:**
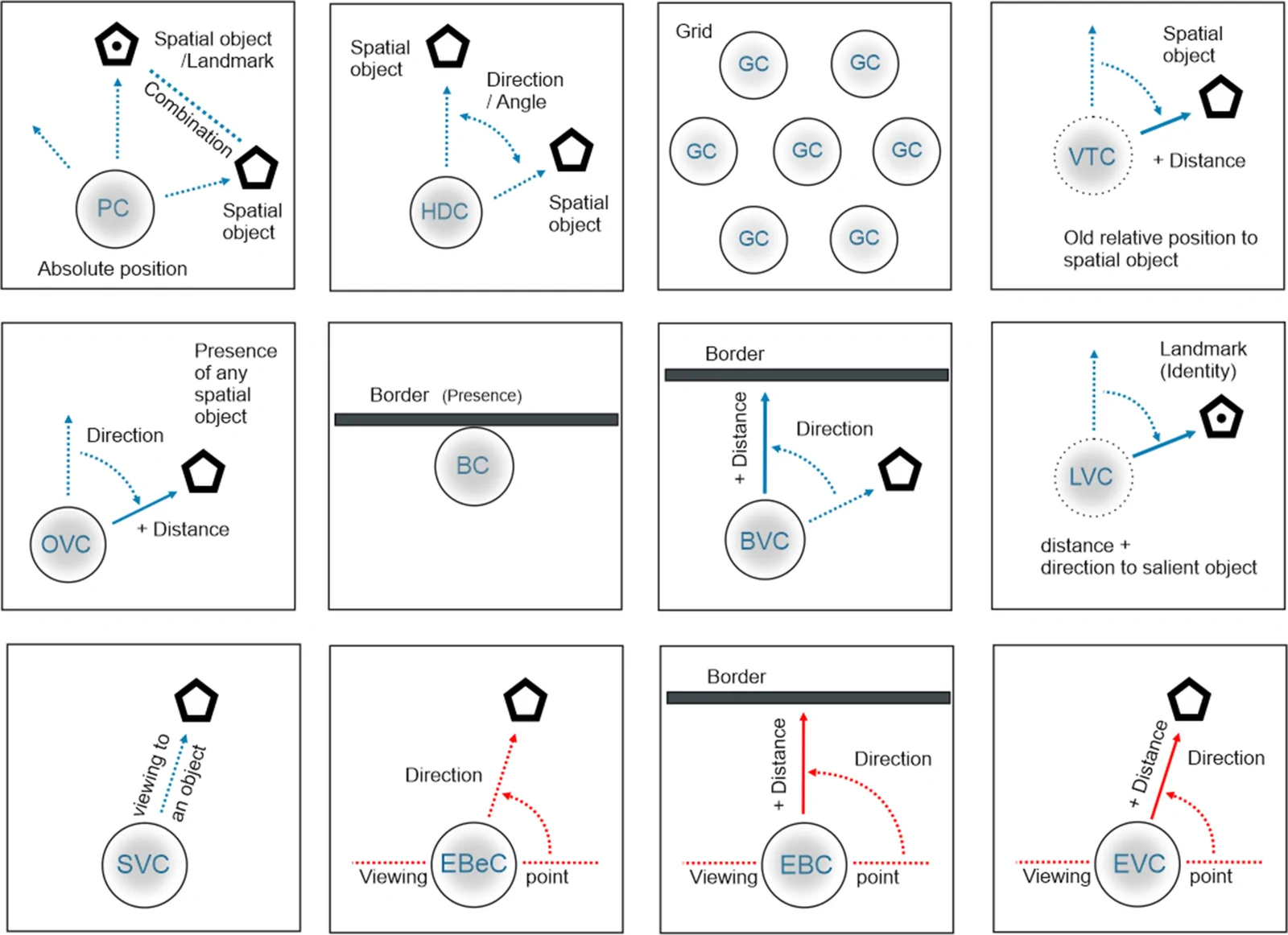
Activity patterns of different spatially tuned cells in response to external (topographic) cues in an environment*; place cells (PC), head direction cells (HDC), grid cells (GC), vector trace cells (VTC), object vector cells (OVC), border cells (BC), boundary vector cells (BVC), landmark vector cells (LVC), spatial view cells (SVC), egocentric bearing cells (EBeC), egocentric boundary cells (EBC), egocentric vector cells (EVC)*

**Fig. 13:**
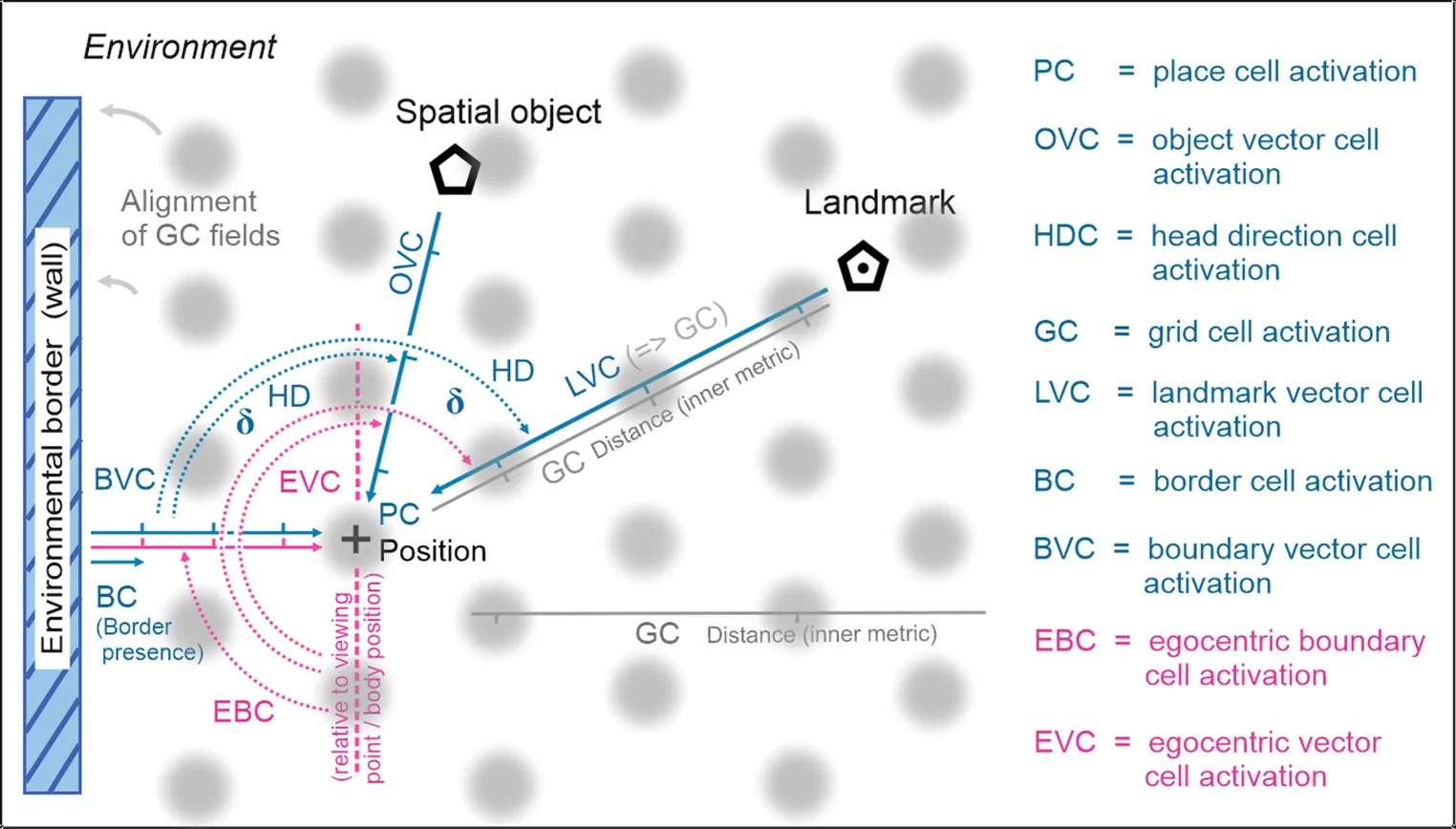
Schematic representation of cell-based encoding of object positions in an environment: *border cell* populations (BCs, BVCs, EBCs, EVCs) represent the boundaries of an environment; *head direction cells* (HDCs) represent directional information anchored to the external environment (independent of body orientation) and enable the encoding of angular distances (δ) between objects; *grid cells* (GCs) provide the basis for internal distance calculation within an environment due to the regular geometry of their firing patterns (grey) and align themselves with environmental boundaries (cardinal axes); *Landmark vector cells* (LVCs) encode distance and direction to salient objects *with identity* (meaningfulness). Object vector Cells (OVCs) encode direction and distance to objects *without identity* based on visual cues (ultimately based on grid cell metric and head direction cell information). In parallel, egocentric vector cells (EVCs) and egocentric boundary cells (EBCs) represent space egocentrically (i.e., depending on body orientation*). Place cells* (PCs) encode one’s own position based on the relative position to other objects (including their identity)

Only some of the neural processes contributing to an allocentric representation of the environment are currently known, particularly regarding the interaction of the involved neural networks (Moser and Moser 2008Moser and Moser 2016). Nevertheless, insights into the properties of spatially tuned cells could provide valuable perspectives on the mechanisms underlying the construction of a cognitive map. Although most findings are based on animal studies, there is increasing evidence for their transferability to humans (Doeller et al. 2010; Kunz et al. 2021). The firing properties of these cells reflect specific aspects of the orientation process and can, at least in part, be linked to external influences (see Fig. 12 and 13).

When an individual enters a new environment, self-motion signals and stable (salient) objects as well as their spatial configurations—contribute to the activation of spatially tuned cells such as *place cells, grid cells,* or *head direction cells* (cf. Knierim et al. 1995; O’Keefe and Conway 1978). The hexadirectional *grid cell* pattern generated by movement signals then aligns with dominant environmental cues, such as boundaries and walls, and becomes stabilized. *Grid cell* activity serves to anchor objects within the spatial geometry of allocentric representations (Hafting et al. 2005). Border cells (BCs) represent information about the spatial limits (boundary identity) of the environment. In parallel, the activity of egocentric border-vector cells (EBVs) likely provides information about the distance between the individual’s position and a boundary during navigation (Lever et al. 2009). Boundary-vector cells (BVCs), on the other hand, encode both distance and direction to an environmental boundary (e.g., a wall) in relation to other objects in the environment, thereby contributing to an allocentric representation (Bicanski and Burgess 2020). Applied to human navigation, this suggests that features such as a hedge, a roadside ditch, or even a simple ground marking may elicit corresponding responses in egocentric and allocentric border cells. The critical input for processing directional information—on which the activation of vector-encoding cells such as EBVs and BVCs depends—likely originates from (allocentric) head direction cells (Bicanski and Burgess 2020). In an environment, these cells respond to specific directions anchored to the location of stable objects (visual landmarks), even when an individual moves through space or changes body orientation. The representation is relative to the environment, i.e., independent of the body’s current orientation (Taube et al. 1990). This provides the basis for processing angular relationships between objects of an environment.

Object vector cells (OVCs), in turn, play an important role in encoding spatial objects within the boundaries of an environment. They represent directional and distance information to spatial objects based on visual inputs (Høydal et al. 2019). The directional information they provide is allocentric within the explored environment. This means that an object (such as a building or tree) may be represented as being located 100 m north of the current position (‑assuming that the individual knows where north is within the environment). Egocentric vector cells (EVCs), in turn, extend the principle to objects from the egocentric perspective (Park et al. 2024). The distance coding of allocentric and egocentric vector cells (BVCs, OVCs, EVCs) is likely grounded in the metric framework provided by grid cell activity (cf. Bicanski and Burgess 2020). Grid cell activity allows a position (e.g., one’s own location) to be anchored relative to surrounding objects within the local spatial geometry.

The combined input of surrounding objects (together with their meanings or identities) and spatial information such as distances and directions enhances the firing activity of the involved cell populations. This activity likely serves as input for the activation of place cells, which encode the individual’s own location (cf. McNaughton et al. 2006; O’Keefe and Conway 1978). For the construction of an allocentric representation of space, this means that different systems in the hippocampus and adjacent brain regions complement one another. Border cell types (BCs, BVCs) provide the basic geometry (i.e., the environmental boundaries) of an area (cf. Bicanski and Burgess 2020), while object vector cells (OVCs, PCs, EVCs) encode individual objects within this geometry, thereby completing the topography. Additional cell populations complement or overlap with these processes. For example, landmark vector cells (LVCs) are likely involved in encoding salient objects. Unlike OVCs, LVCs encode the identity of an object or landmark (cf. Deshmukh and Knierim 2013). These processes are thought to operate interactively and recursively, such that during movement through an environment continuous corrective feedback is provided (cf. McNaughton et al. 2006).

The spatial parameters encoded by spatially tuned cells—in particular position, distance, and direction—can be conceptually related to graph-based models of navigation. Spatially tuned cells encode fundamental aspects of spatial orientation and thereby provide a neural representation of locations, movement directions, and distances within the environment. A theoretical framework that integrates these different sources of spatial information is the cognitive graph model proposed by Chrastil and Warren (2014). In this framework, spatial knowledge is represented as a network of locations (nodes) connected by traversable paths (edges) that are supplemented with local metric information such as distances and angles. Within this architecture, grid cells may provide metric distance information, while head-direction cells encode angular orientation.

These signals establish a fundamental spatial reference structure upon which additional neural representations can be constructed. Neurons that encode positions relative to external reference points—such as object vector cells, landmark vector cells, and boundary vector cells—integrate distance and directional signals to represent the position of objects, landmarks, or environmental boundaries relative to the observer. Within this framework, place cells can be interpreted as representing discrete locations (nodes) within the spatial graph, whereas grid cells provide metric information about distances between these locations (Fig. 13). Head-direction cells encode movement direction, while border cells signal environmental boundaries that constrain the topological structure of possible routes.

A key step in building an “inner map” is converting egocentric (body-centered) information into allocentric (world-centered) representations that support orientation beyond the current viewpoint. From a cartographic perspective, egocentric “polar coordinates” of objects must be transformed into “Cartesian coordinates” to allow universal retrieval of spatial information that is not tied to a specific viewpoint (cf. Kunz et al. 2021, for humans). Allocentric (map-based) representation is grounded in fixed, external reference points in the environment, such as landmark positions or boundaries (Kozhevnikov and Puri 2023; Epstein and Vass 2014). It enables, for example, linking the start and goal of a route within the same spatial grid or detecting potential shortcuts. In contrast to egocentric representations, which use the body with individual movement and bodily rotations as a reference perspective (Kozhevnikov and Puri 2023), allocentric representations of the environment are independent of one’s current body position or immediate location (Buzsáki and Moser 2013; Rolls 2022). Allocentric representations therefore play a decisive role in orientation performance (cf. Zhou et al. 2023).

However, during navigation, sensory information (e.g., viewing a landmark) is initially processed in an egocentric frame of reference (Long et al. 2025). Egocentric spatial information is encoded by *egocentric bearing cells* (EBeCs) (Kunz et al. 2021) and *egocentric vector cells* (EVCs) (Alexander and Nitz 2015; Park et al. 2024). The firing activity of EBeCs predominantly represents the direction from the individual’s position to a single object or landmark (but not a boundary). EVCs, by contrast, encode not only the direction but also the distance to an object or salient corner of the environment. Like OVCs and BVCs, they therefore represent “vectors”.

The flow of information must also be able to run in the inverse direction (i.e., from the internal map to the real world) when allocentrically stored representations are retrieved for goal-oriented navigation and planning (Byrne et al. 2007). During recall from spatial working memory, spatial information must be transformed from the allocentric cognitive map back into an egocentric reference frame, so that navigation can proceed relative to the individual’s current position in space (Bicanski and Burgess 2018; Hinman et al. 2019; Long et al. 2025). Successful orientation therefore depends on the use of both allocentric (world-centered) and egocentric (self-centered) representations (Buzsáki and Moser 2013).

Thus, successful navigation typically may emerge from an interaction between allocentric and egocentric reference frames. Egocentric orientation processes—such as maintaining orientation relative to landmarks, heading direction, or self-motion cues—can play a dominant role in many navigation contexts and may support flexible wayfinding even in the absence of map-based representations (e.g., Thorndyke and Hayes-Roth 1982). Consequently, the “neurocartographic” perspective proposed here aims to support both egocentric and allocentric spatial encoding by structuring spatial information in ways that align with known neural mechanisms of navigation.

## External Influences on the Construction of a Cognitive Map

Recent studies point to a strong relation between *grid cell* activity and spatial memory performance (Gil et al. 2018; Ying et al. 2022). It can therefore be assumed that higher firing rate of egocentrically and allocentrically tuned spatial cells generally contributes to an improved perception of the environment. This is also supported by studies conducted in humans (e.g., Doeller et al. 2010; Kunz et al. 2015; Navarro Schröder et al. 2020). Maidenbaum et al. (2018) and Killian and Buffalo (2018) found evidence that fMRI signals—believed to specifically reflect *grid cell* firing activity (“grid-cell-like representations”) in the human brain (Doeller et al. 2010)—are closely linked to navigation performance. This raises the question of whether the activity of allocentric spatial cells can be influenced—or even controlled—by external (geographic) stimuli.

Neuroscientific research has identified a range of external environmental cues that selectively affect the firing activity of different spatially tuned cells (Table 2). These cells do not fire solely because of intrinsic neural circuitry, but also directly in response to environmental cues (Buzsáki and Moser 2013; Hafting et al. 2005; Navarro Schröder et al. 2020; cf. Doeller et al. 2010 for humans). Environmental boundaries and especially distal landmarks hold a particularly prominent position to enhance orientation capabilities (e.g., for head direction cells; cf. Mehlman and Taube 2018).

**Table 2:** Examples of the influence of external cues on the activity of spatially tuned cells (mostly based on animal experiments)

Cell type	Cell activity (in 3D space)	Triggering external input/cue
Place cells/Landmark vector cells/ Object vector cells	Activity based on perception of salient environmental stimuli	Stable landmarks, (new) objects, multisensory feedback from visual, auditory, tactile, and olfactory cues (e.g., distinctive shapes, sizes, colors); object meaning (relevance) (Kentros et al. 2004; Komorowski et al. 2009; Savelli et al. 2017; Scaplen et al. 2014)
	Modulation of activity as a result of motivation/task/attention (PC/LVC)	Prospect of success/reward (Krishnan et al. 2022; cf. Gauthier and Tank 2018)
Head direction cells	Activity stabilized perception of salient environmental stimuli	Stable (primarily visual) landmarks, self-motion information (Mehlman and Taube 2018; Goodridge et al. 1998)
Grid cells	Activity resulting from self-motion/alignment with environment/anchoring	Environmental boundaries, landmarks, spatial axes, e.g., cardinal directions, meaning (social cues) (Hafting et al. 2005; Xu et al. 2022)
	Stabilization of firing patterns	Self-motion (vestibular feedback), stable landmarks; symmetrical environments; environmental constancy; repeated exploration (experience). (Krupic et al. 2015)
Border cells/Boundary vector cells/Egocentric boundary cells	Activity following perception of environmental boundaries	Visual cues (walls), optic flow, (Kunz et al. 2021; Raudies and Hasselmo 2012; Yang et al. 2023)

However, external cues are not confined to visual input; auditory, tactile, and olfactory stimuli can likewise modulate the activity and stability of spatially tuned cells. Moreover, context-dependent factors—such as motivational states associated with navigation (e.g., expectation of reward or success) (Krishnan et al. 2022; cf. Gauthier and Tank 2018) or socially mediated object meaning (Xu et al. 2022)—further influence these neural representations.

## “Neurocartographic” Implications

Spatial orientation arises from activity patterns of numerous interconnected and interdependent cell populations in the hippocampus, the entorhinal cortex and adjacent regions. Different types of spatially tuned cells represent only partial aspects of spatial information, but their coordinated interplay ultimately supports the construction of an allocentric spatial representation—an “inner map”—of the environment. The properties of the spatially tuned cells discussed here not only illuminate the neural mechanisms underlying orientation processes but also show how their activity can be modulated by external influences. Such environmental cues are particularly important, as stabilizing the firing patterns of spatially tuned neurons has been shown to facilitate effective navigation (Navarro Schröder et al. 2020; Gil et al. 2018).

From a cartographic perspective, this raises the prospect of leveraging external cues to design media-based approaches that enhance human orientation performance (Dickmann et al. 2024). When reading a map (e.g., to memorize a route), users visually trace the course of roads in relation to boundaries and landmarks (Keil et al. 2018, 2020a), a process that may involve the mental simulation of movement through the depicted environment. For example, visually emphasized boundaries or spatial partitions in a map may correspond to environmental borders that are known to modulate the activity of border cells. Similarly, the depiction of spatial distances, routes, and spatial layouts may support the formation of metric spatial representations that could potentially engage grid-cell-related coding mechanisms. Such cognitive operations may partially recruit neural systems involved in spatial navigation, for example through the processing of visually represented boundaries or through mental simulation of spatial movement (Horner et al. 2016). Consequently, specific design features of spatial information displays may support spatial encoding by aligning visual map elements with neural mechanisms underlying spatial representation.

Well-designed external cues processed in the hippocampal-entorhinal network, may enhance the activity of spatially tuned cells. VR/AR-based visualization techniques, for instance, could accentuate landmarks within the visual field or integrate supplementary cues into head-up displays for vehicular navigation (Dickmann et al. 2021). Even in conventional two-dimensional maps, graphical structures could be purposefully designed to align with the activity profiles of spatially tuned cell populations to support the construction of an inner map.

The pronounced sensitivity of *place cells* and *head direction* cells to salient environmental features underscores the central role of landmarks in navigation and highlights the need to systematically identify and emphasize suitable landmarks (Cheng et al. 2022; Keil et al. 2025, 2020a; Li et al. 2014). Such features can also be visually accentuated in virtual environments (Credé et al. 2020). For the stabilization of *grid cells*, by contrast, the alignment of firing fields along spatial axes such as terrain edges, rivers, or mountain ridges appears critical. The metric structure of the grid could then provide a robust allocentric reference framework into which spatial objects can be more efficiently integrated in terms of distance and direction. The geometry of environmental boundaries can define the principal axes of an allocentric reference frame, to which grid-cell activity may become aligned (Krupic et al. 2015). Analogously, the cardinal axes and structural features depicted on maps may provide an external reference frame that supports the construction of internal spatial representations.

Initial empirical studies investigating the effects of environmental boundaries, particularly cardinal axes, suggest that their presence can measurably improve spatial navigation performance (Korte et al. 2024; Navarro Schröder et al. 2020). However, further research is needed to identify the specific characteristics of external cues that most effectively promote the stability of firing patterns in spatially tuned cells. To address this challenge, a dedicated “neurocartographic” research agenda is required that systematically investigates how environmental cues can be designed to strengthen allocentric spatial representations and, consequently, enhance human navigation performance (cf. Hilton et al. 2025; Kapaj et al. 2024). Integrating controlled navigation experiments with functional magnetic resonance imaging (fMRI) would make it possible to relate behavioral improvements to underlying neural activity, thereby providing indirect evidence for the engagement of different spatially tuned cell systems. A supplementary eye-tracking study (recording participants’ eye movements) could provide valuable insights into which environmental features, landmarks, boundaries, or map elements participants attend to during navigation, thereby helping to identify the visual information that may contribute to the formation and stabilization of internal spatial representations. Furthermore, consecutive VR-based spatial navigation studies could be used to investigate the effects of identified spatial cues on navigation performance with increased ecological validity (Keil et al. 2026). These methods could provide a mechanistic foundation for evidence-based “neurocartographic” principles, allowing (represented) external cues to be designed in ways that selectively support the neural processes underlying human navigation.

## Data Availability

All data supporting the findings of this work are included in the article.
